# Infectivity of Zika virus on primary cells support tree shrew as animal model

**DOI:** 10.1080/22221751.2018.1559707

**Published:** 2019-02-04

**Authors:** Li Zhang, Zhi-Li Shen, Yue Feng, Dao-Qun Li, Na-Na Zhang, Yong-Qiang Deng, Xiao-Peng Qi, Xiao-Mei Sun, Jie-Jie Dai, Chun-Guang Yang, Zi-Feng Yang, Cheng-Feng Qin, Xue-Shan Xia

**Affiliations:** a Faculty of Environmental Science and Engineering, Kunming University of Science and Technology, Kunming, People’s Republic of China; b Faculty of Life Science and Technology, Yunnan Provincial Center for Molecular Medicine, Kunming University of Science and Technology, Kunming, People’s Republic of China; c State Key Laboratory of Pathogen and Biosecurity, Beijing Institute of Microbiology and Epidemiology, Beijing, People’s Republic of China; d Key Laboratory of Animal Models and Human Disease Mechanisms, Kunming Institute of Zoology, Chinese Academy of Sciences, Kunming, People’s Republic of China; e Center of Tree Shrew Germplasm Resources, Institute of Medical Biology, Chinese Academy of Medical Science and Peking Union Medical College, Kunming, People’s Republic of China; f State Key Laboratory of Respiratory Disease, National Clinical Research Center for Respiratory Disease, First Affiliated Hospital of Guagnzhou Medical University, Guangzhou, People’s Republic of China

**Keywords:** Zika virus, tree shrew, primary cells, tropism, infectivity

## Abstract

Zika virus (ZIKV) is a mosquito-borne flavivirus that caused the public health emergency. Recently, we have proved a novel small animal tree shrew was susceptive to ZIKV infection and presented the most common rash symptoms as ZIKV patients. Here we further cultured the primary cells from different tissues of this animal to determine the tissue tropism of ZIKV infection *in vitro*. The results showed that the primary cells from tree shrew kidney, lung, liver, skin and aorta were permissive to ZIKV infection and could support viral replication by the detection of viral specific RNA intra- and extra-cells. In comparing, the skin fibroblast and vascular endothelial cells were highly permissive to ZIKV infection with high releasing of active virus particles in supernatants proved by its infectivity in established neonatal mouse model. The expressions of ZIKV envelop and nonstructural protein-1, and the effects and strong immune response of primary tree shrew cells were also detected followed by ZIKV infection. These findings provide powerful *in vitro* cell-level evidence to support tree shrew as animal model of ZIKV infection and may help to explain the rash manifestations *in vivo*.

## Introduction

Zika virus (ZIKV) is a positive-sense single-stranded RNA virus of *Flaviviridae* family, *Flavivirus* genus. ZIKV was originally identified in a sentinel rhesus monkey in the Zika Forest of Uganda in 1947 and thereafter remained a silent spreading for over half a century without catching much attention [1]. Surprisingly, ZIKV was re-emerged in 2016 in Brazil and then rapidly spread worldwide [2]. ZIKV infection in human beings normally results in a self-limiting febrile illness with common symptoms such as rash, conjunctivitis, and joint pain [3]. And it’s also associated with some neurologic disorders including foetal microcephaly, brain anomalies, spontaneous abortion and Guillain-Barre syndrome (GBS) [4,5]. Currently, both the worldwide transmission and deleterious clinical outcomes of ZIKV infection have triggered a global public health emergency and WHO has recently declared a public health emergency for Zika fever [6].

In order to elucidate the pathogenesis mechanisms of ZIKV infection and host immune response, and further to develop antiviral drugs and vaccines, various animal models have been established. Among them, Non-human primates (NHPs) were the ideal models. ZIKV-infected NHPs may develop viremia [7,8]. The Central nervous system (CNS) damage, and shedding virus in different tissues including placenta, foetal brain and liver and maternal brain, eyes, spleen, and liver [9]. However, rash of the typical manifestation is mild and only developed in few rhesus macaques [7,10]. Besides, a variety of knockout or antibody treatment mice also established ZIKV infection and recapitulated many features of human diseases, like foetal abnormalities and microcephaly [11–16]. But, the adult immunocompetent mice did not establish any clinical disease and few or no virus was detected in wild-type (WT) mice like C57BL/6, Swiss Webster, BALB/c, and CD-1 [17–19]. Nevertheless, each of these models has limitations, the high cost of macaque studies, and chiefly poor ZIKV replication in mice. Thus, there is a continue need for new animal model that can recapitulate disease features of ZIKV infection in humans.

Moreover, lots of *in vitro* investigations were also performed to address the virus infectivity and pathogenesis *in vivo*. A broad range of human and nonhuman cell lines has shown different susceptibility to ZIKV infection [20]. Comparing with cell lines, primary cell may present more similar characteristics of viral infection and pathogenicity in *vivo*. The human primary cells from skin, testis, brain, placenta, kidney and retina, as well as the immune cells were permissive to ZIKV infection [21].

Tree shrew is a squirrel-like and rat-sized mammal widely distributes in Southeast Asia and south-west China. This animal is much more closely related to humans than the rodents [22,23] and has proved to be susceptible to variety of human viruses, including hepatitis B virus [24], hepatitis C virus [25], hepatitis E virus [26] and herpes simplex virus [27]. As for ZIKV, we previously challenged the tree shrew with ZIKV via subcutaneous and found that tree shrew was susceptive to ZIKV infection. Remarkably, the infected tree shrew recapitulated some typical clinical symptoms like, skin rash, cutaneous inflammation and transient viremia. These results indicated that tree shrew can be served as a potential immunocompetent small animal model to study ZIKV infection [28]. In this study, we performed *in vitro* ZIKV infection on different tree shrew primary tissue cells and tested for the presence of viral RNA, infectious virus, antigen expression and immune responds. These findings may provide powerful in vitro cell-level evidence to support tree shrew as animal model of ZIKV infection.

## Results

### Susceptibility of different tree shrew primary cells to ZIKV infection

To examine the susceptibility of primary cells of tree shrews to ZIKV infection *in vitro*, we obtained different primary cells from corresponding tissues of tree shrew, including primary skin cells (TSDF), primary thoracic aorta cells (TSVE), primary kidney cells (TSKC), primary lung cells (TSEL) and primary liver cells (TSHC). These primary cells, along with human origin cell lines, HUVEC (human umbilical cord), HFF-1 (human foreskin fibroblasts), HEK293 (human foetal kidney), HEL (human embryonic lung fibroblasts), Huh7.5.1 (human hepatocellular carcinoma), were infected with ZIKV at an MOI = 1. The ZIKV RNA and specific strands of viral RNA in supernatants and in cells at various time points of post-infection were determined by quantitative RT-PCR.

**Figure 1.**
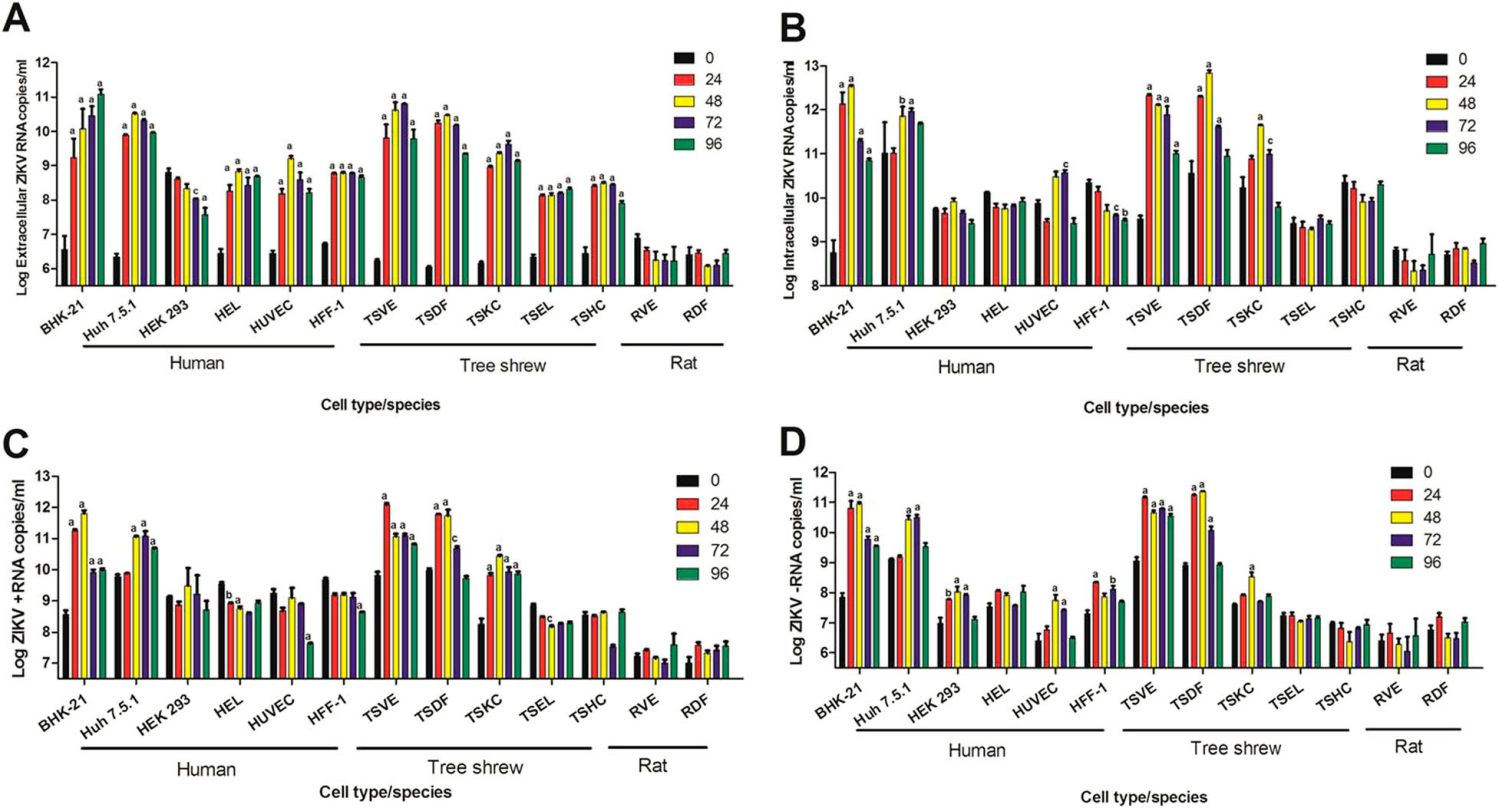
Susceptibility of primary cells to ZIKV infection. Different cell infected with ZIKV (MOI = 1) and analysed at different time of post-infection. Virus RNA loads in extracellular (A) and intracellular (B), cellular positive (C) – and negative strand (D) ZIKV RNA were detected quantitatively by real-time RT-PCR. All experiments were done in triplicate. The mean viral loads on hours 24, 48, 72, 96 were compared with the baseline viral load on hour 0 (1 h post-ZIKV inoculation). All calculations were based on log-transformed viral loads. *P*-values of <.001, <.01, and <.05 were labelled as a, b, c.

As the accepted host cell, BHK-21 showed a gradually increase of viral RNA in its supernatant over time, peaked to 10^11.09^ RNA copies/ml at 96 h of post infection (hpi) (Figure 1(A)). Except for HEK293, other human cells had a significantly increase in the period of ZIKV infection, particularly Huh7.5.1 had an apparent increase and could reach 10^10.5^ viral copies/ml. Of note, the viral load in supernatants of all the tree shrew primary cells from various tissues also increased overtime. Of them, TSVE and TSDF had significantly higher level of viral RNA than the other cells, and peaked to 10^10.79^ and 10^10.45^ RNA copies/ml at 72 and 48 hpi, respectively (Figure 1(A)).

The level of intracellular ZIKV RNA had a similar trend. BHK-21, Huh7.5.1 TSVE and TSDF increased obviously over the infection time course. Notably, TSVE and TSDF quickly reached 10^12.23^ and 10^10.34^ RNA copies/ml in cells at 24 hpi (Figure 1(B)). Since ZIKV is positive-stranded RNA virus, the present of negative-strand RNA is an important indicator of ZIKV replication within the infected cells. The result of negative-strand RNA showed that all cells, except for TSEL and TSHC, had a significantly increased (*p* < .01). In particularly, TSVE and TSDF had a higher level compared with other human cells and other primary cells from tree shrew (Figure 1(D)). Meanwhile, the level of negative-strand RNA in each cells were lower than that of positive-strand RNA (Figure 1(D)) as expected. Thus, we considered TSVE and TSDF as the best permissive to ZIKV replication.

In parallel, we take SD rat as control animal, the rat primary aorta cells (RVE) and skin cells (RDF) were obtained using the same method, and then infect with the same ZIKV stocks. The quantitation of ZIKV RNA no matter in supernatant or in cells showed no evidence of virus infection and replication in primary skin and aorta cells from rat in comparing with that of TSVE and TSDF (Figure 1).

### The expression of ZIKV proteins in primary TSVE and TSDF cells

**Figure 2.**
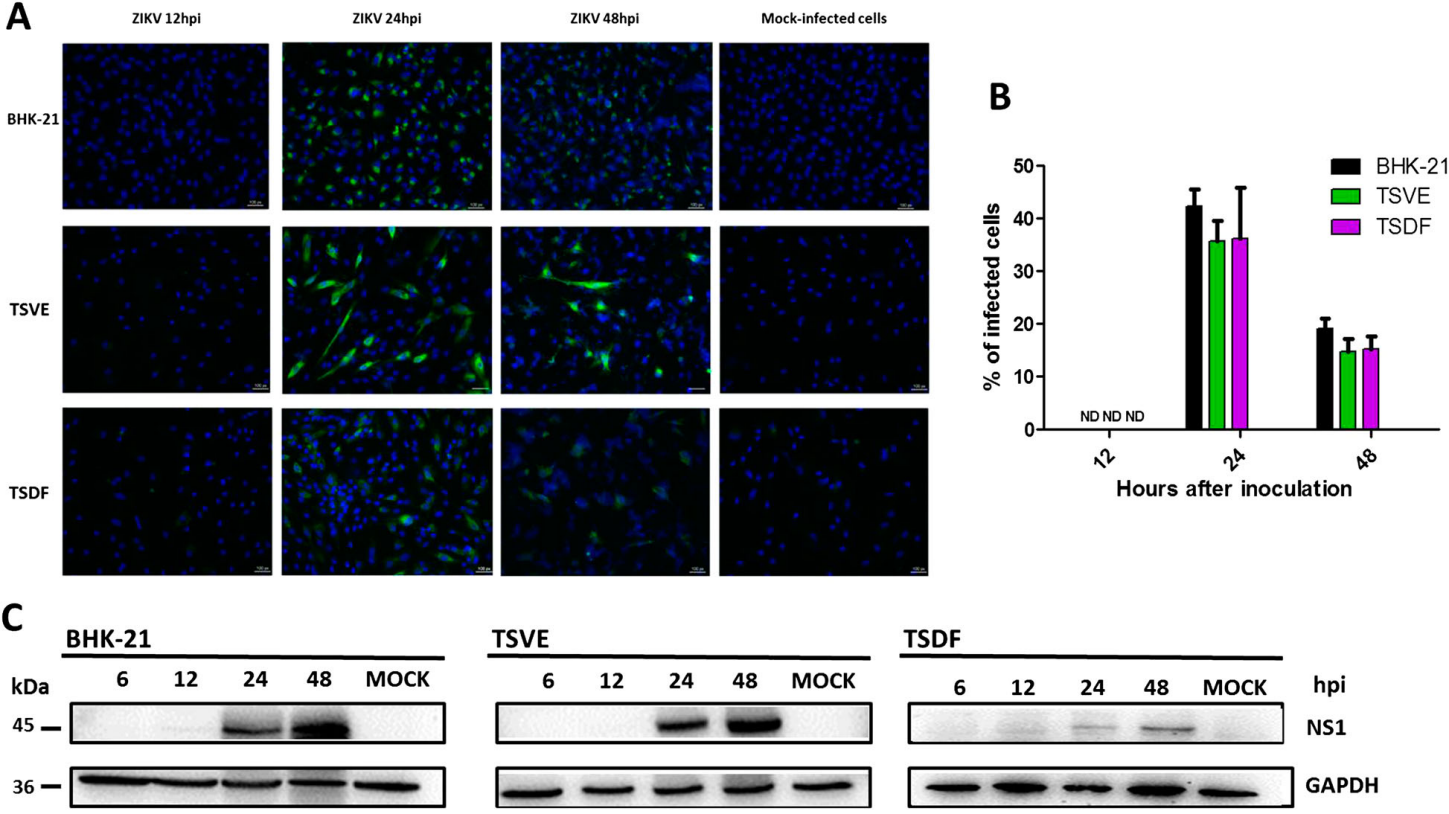
Expression of ZIKV proteins in the primary tree shrew skin and artery cells. (A) Immunolocalization of ZIKV E protein in cells at different time of ZIKV post-infection. Positive viral proteins were shown in green and DAPI in blue. (B) The graph represents the mean ± standard deviation of the infection percentage (%). (C) Cells were exposed to ZIKV at the indicated times, and NS1 protein levels were detected by Western blotting, and GAPDH as a control. Data are representative of three independent experiments. All scale bar: 100 μm.

To further confirm the susceptibility of primary TSVE and TSDF to ZIKV, the expression of ZIKV proteins was detected. The presence of viral envelope proteins in cells was evaluated by indirect immunofluorescence assay (IFA) immune-stained with the human convalescent serum from ZIKV patient. As a result, the viral envelope protein was detected and reached the highest number of infected cells, about 42.3% of BHK-21, 35.8% of TSVE and 36.3% of TSDF at 24 hpi, respectively. In contrast, no staining was detected in mock-infected cells (Figure 2(A)). Consistent with the presence of envelope proteins in primary cells, we observed ZIKV NS1 protein, which was participated in the viral replicate process, was also appeared from 24 to 48 hpi (Figure 2(B)), but TSDF ZIKV-NS1 expression was much less prominent than that in TSVE. Overall, ZIKV infected tree shrew primary TSVE and TSDF were viable allowing the viral protein express stably to guarantee the virus propagation.

### Kinetic of ZIKV replication and morphological changes in primary TSVE and TSDF cells

**Figure 3.**
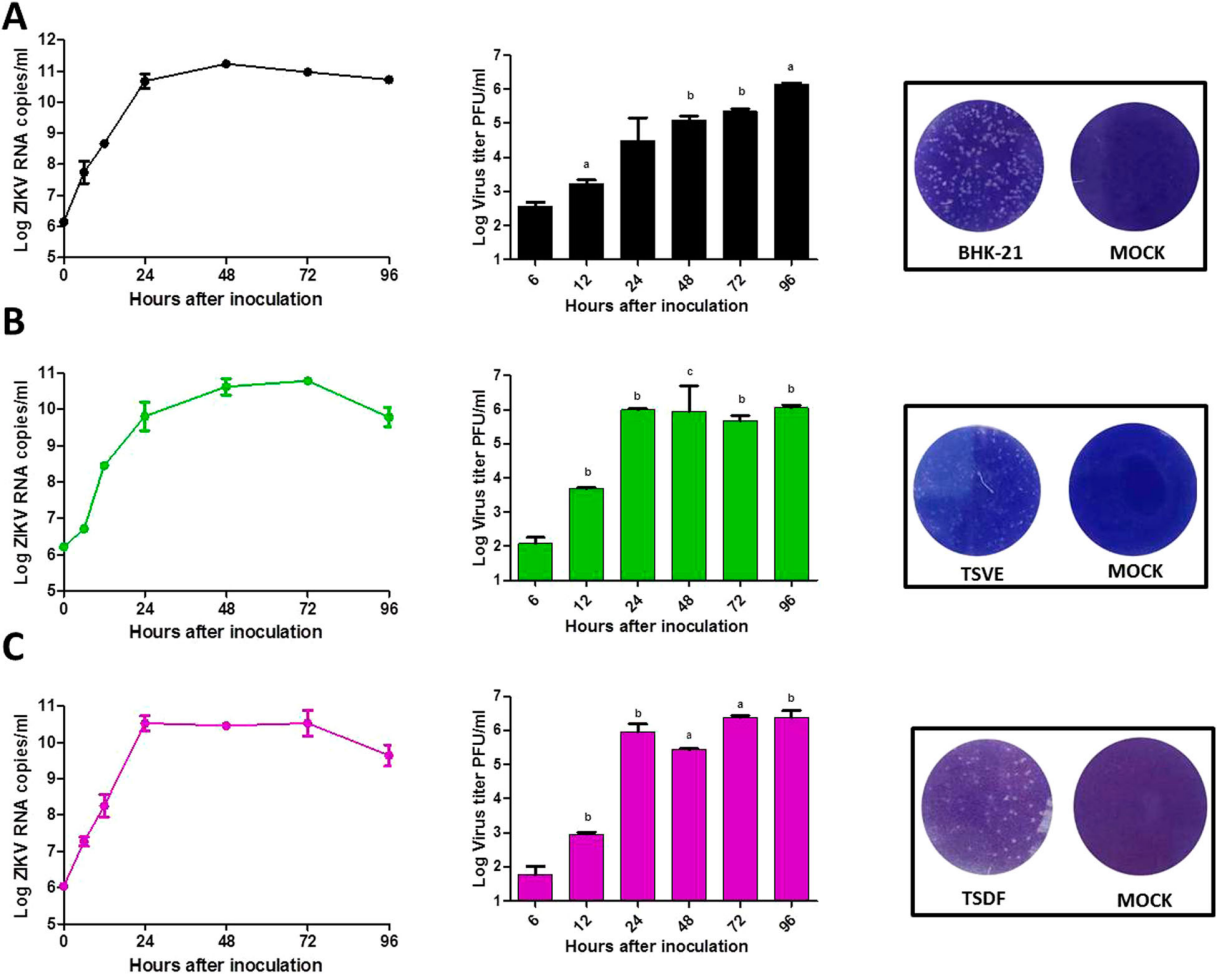
Kinetic of ZIKV replication in the Primary skin and aorta cells. The viral RNA copies was determined by real-time RT-PCR (left), ZIKV viral titer in supernatants of infected cells was determined by plaque assay (middle) and the representative photographs of plaques using supernatants from above cells (right). (A), (B) and (C) were the results of BHK-21, TSVE and TSDF, respectively. *P*-values of <.001, <.01, and <.05 were labelled as a, b, c.

**Figure 4.**
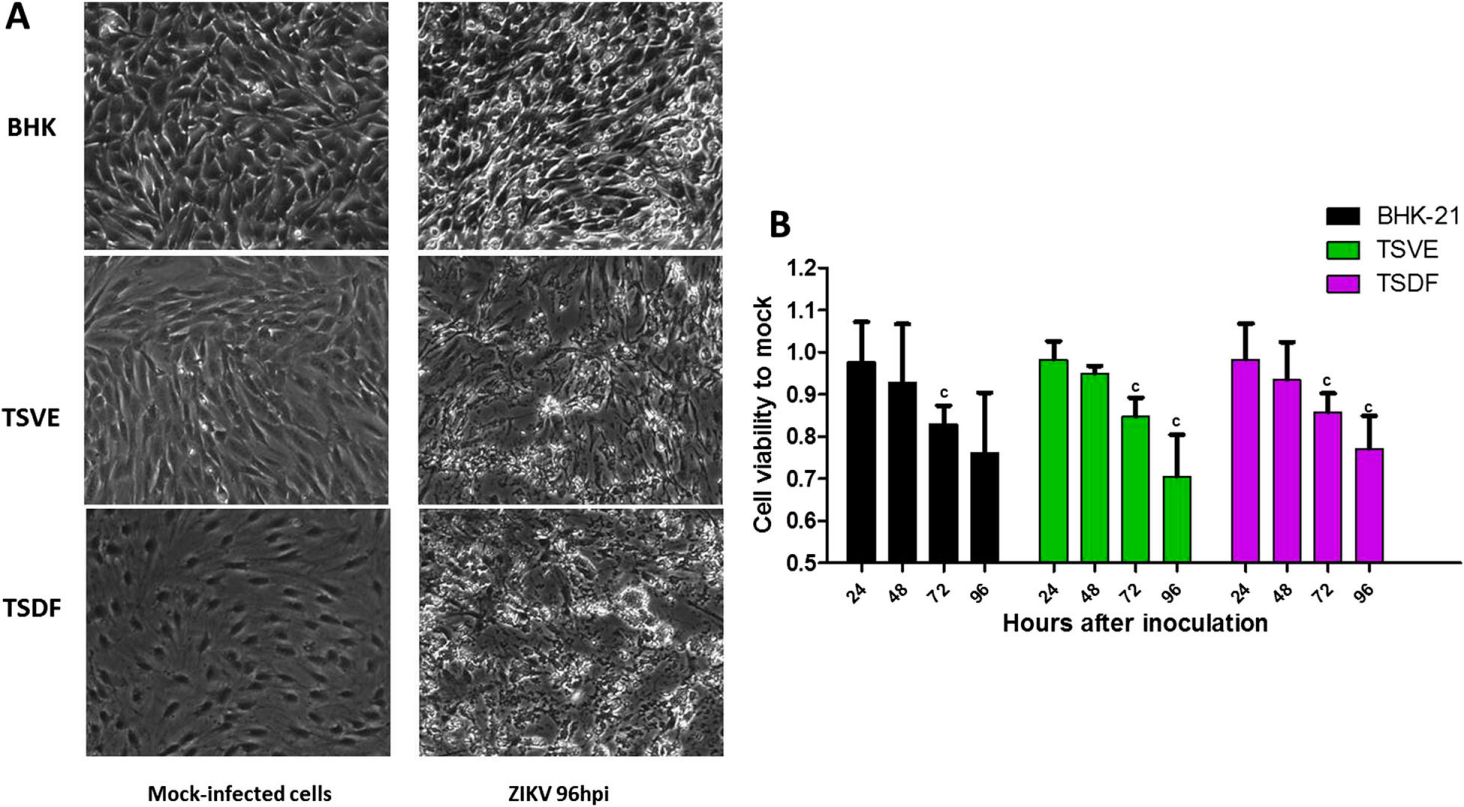
Cytopathic effects of ZIKV infection. (A) Subconfluent monolayers of cells were infected at an MOI of 1 with ZIKV or mock-infected (control). Cells were micro-observed by using inverted phase-contrast microscope (original magnification, ×20). (B) The cell viability was calculated by comparison to mock-infected cells at the corresponding time point after ZIKV infection. *P*-values of <.001, <.01, and <.05 were labelled as a, b, c.

After ZIKV infection on TSVE and TSDF, the viral RNA in supernatants quickly reached a high level (Figure 1(A)) with a linear increase of viral load. In addition, we evaluated the ability of these cells to produce viral progeny using a standard plaque assay. Similar to the results obtained with BHK-21 cell line, primary TSVE and TSDF could produce infectious viral particles at a time-dependent manner (Figure 2). The viral loads had a small decrease at 96 hpi, which mostly because the majority of cells were dead (Figure 3). Compared with the mock cell, ZIKV infection induces notable cytopathogenic effects (CPE) in TSVE and TSDF at 96 hpi (Figure 4(A)). Actually, cytolysis, cell shrinkage, foci of cell destruction and a large number of detached cells in the supernatants were showed at this time point. Moreover, the viability of the three cells began to decline after ZIKV infection (Figure 4(B)). These data collectively indicated that TSVE and TSDF can support robust ZIKV infection with significant diminishing cells viability.

### Infectivity of produced virus by the ZIKV-infected primary TSVE and TSDF cells

**Figure 5.**
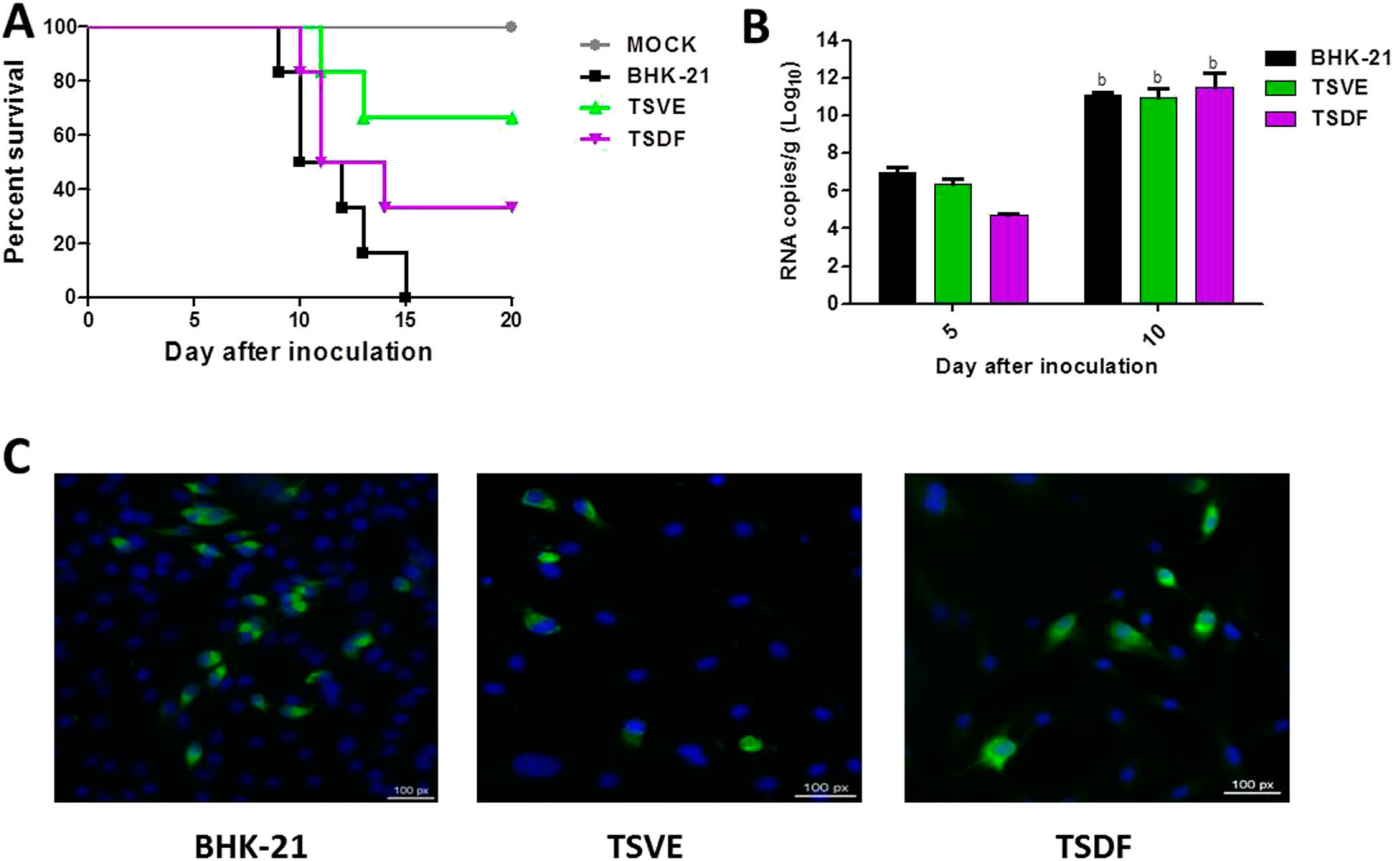
Infectivity of progeny virus. (A) Survival curve of the ZIKV-infected neonatal one-day-old suckling BALB/C mice. Groups of mice were inoculated with 10^3^ PFU of the supernatants from the ZIKV-infected BHK-21 (*n* = 6), TSVE (*n* = 6) and TSDF (*n* = 6) by the intracerebral route. PBS was included as mock control (*n* = 5). (B) Viral RNA loads in the brains from the ZIKV-infected neonatal mice were determined by real-time RT-PCR at 5 and 10 dpi. (C) The supernatants of ZIKV-infected re-infection the fresh cells and then detected ZIKV E antigens by immunofluorescence at 24 hpi. Positive viral antigens were shown in green, and DAPI in blue. All scale bar: 100 μm.

To further verify whether the primary cells of tree shrew could produce the infectious virus, the supernatants of ZIKV infected BHK-21, TSVE and TSDF were used to inoculate the established neonatal mouse model [29]. The results showed that the positive control mice infected with the supernatants of BHK (*n* = 6) developed typical neurological symptoms and all died at 9–15 dpi. Of note, the mice infected with the supernatants of TSVE (*n* = 6) and TSDF (*n* = 6) exhibited the distinct paralysis of hind legs range from 10 to 14 dpi, and showed 33.3% (2/6) and 66.7% (4/6) mortality rate respectively within 20 days (Figure 5(A)). However, the mock group (*n* = 5) healthy and grow normally in the experiment period. Additionally, the capacity of ZIKV replication in brains was also studied, the infection resulted in a significant increase of ZIKV RNAs at 5 and 10 dpi, which presents a process of active viral replication in *vivo* (Figure 5(B)).

Next, we performed a test *in vitro* to confirm the presence of infectious ZIKV′naive BHK-21, TSVE and TSDF cells were inoculated with the supernatants, and the presence of viral envelope antigens was evaluated by immunofluorescence at 24 hpi. As Figure 5(C) showed, the three cells could express ZIKV envelop protein. Collectively, these results suggested that the ZIKV-infected primary tree shrew cells could release infectious virus.

### The cytokine expression within primary tree shrews cells in response to ZIKV infection

**Figure 6.**
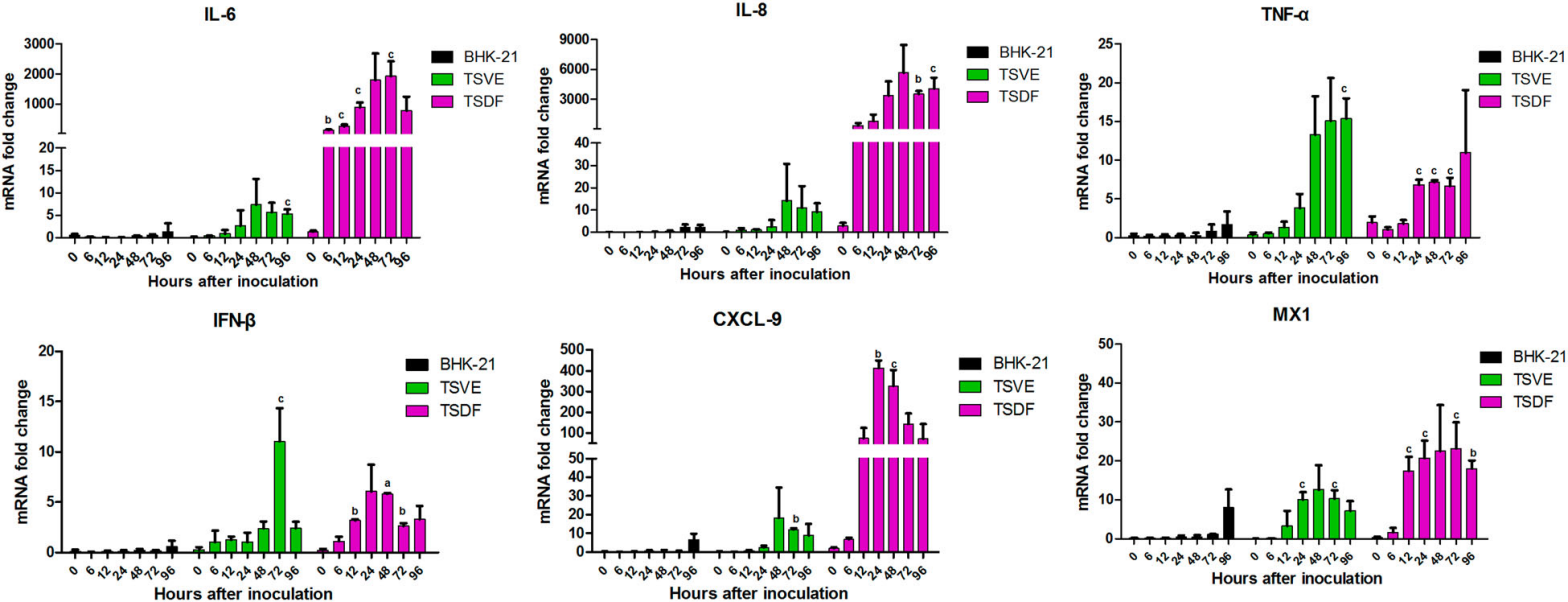
ZIKV induces an innate antiviral response in the primary tree shrew skin and artery cells. Primary cells were inoculated with ZIKV (MOI = 1), and mRNA levels were quantified by using real-time RT-PCR. Results are expressed as the fold induction of transcripts in ZIKV-infected cells relative to those in mock-infected cells. Data are representative of three independent experiments, each performed in duplicate (error bars represent SEM). *P*-values of <.001, <.01, and <.05 were labelled as a, b, c.

In order to determine whether ZIKV induces an innate antiviral immune response in the permissive primary cells, we kinetically analysed the key antiviral immunity-related cytokines genes expression changes in ZIKV-infected cells. For BHK-21, the selected cytokines had no significant change in expression between mock- and ZIKV-infected cells (Figure 5). Conversely, tree shrews primary TSVE and TSDF induced strong antiviral response. TSVE moderately up-regulated the mRNA level of IL-6, IL-8, TNF-α, IFN-β, CXCL9 and MX1 over the infection time. However, the levels of multiple inflammatory cytokines, such as IL-6, IL-8 and TNF-α, were significantly elevated as soon as 6 hpi. The expression of CXCL9, which recruiting circulating leukocytes to inflammatory sites, was highly induced from 12 to 96 hpi. Moreover, the interferon-stimulated genes (ISGs) MX1 were also readily up-regulated. Thus, these results demonstrate that TSVE and TSDF were capable of generating a strong innate immune response to ZIKV infection (Figure 6).

## Discussion

Although ZIKV infection may result an increase in congenital and Guillain-Barre syndromes (GBS), most of infected adults develop mild symptoms, such like fever, rash, arthralgia and conjunctivitis. Recently, we have successfully recapitulated typical dermatological manifestations and viremia that showed in most of patients on the novel tree shrew animal model. Thus, characterizing ZIKV tissues tropism for tree shrew *in vitro* is helpful for further understand the pathophysiology of Zika fever and provides a basis for the development of antiviral drugs by using a relevant cell type.

To determine the extent of primary cells of tree shrew by ZIKV infection, we isolated and cultured primary cells at low passages (passage number < 4) from thoracic aorta (TSVE), skin (TSDF), kidney (TSKC), lung (TSEL), and liver (TSHC). Meanwhile, we obtained human cell lines from the corresponding tissues. The results showed that virus RNA in supernatants of inoculated BHK-21, Huh7.5.1, HUVEC, TSVE and TSDF was significantly increased. The similar trend of virus RNA changes within intracellular was also observed. Notably, the negative-strand RNA, which represents ZIKV replication, reached a markedly high level in TSVE and TSDF. Thus, the human origin cell lines Huh7.5.1 and HUVEC could actively supported ZIKV infection and viral replication, which consistent with the previous research [19,20]. For the primary cell from different tissues of tree shrew, only aorta (TSVE) and skin (TSDF) primary could support ZIKV infection and virus replication cells, which may explain the high rate occurrence of cutaneous rash in tree shrew infected by subcutaneous needling of high titer ZIKV. In comparison no evidence for ZIKV infection in primary rat aorta (RVE) and skin (RDF) cells was observed by using the same inoculation and detection procedure. These results commendably supported that the tree shrew could be taken as a suitable and effective small animal model of Zika fever which particularly display rush mimicking the magnification of patients.

Pathophysiologically, skin is the first tissue for ZIKV infection comes via mosquito biting and then the virus gets into human bloodstream to facilitate its dissemination. For cultured human cells, ZIKV could infect a variety of cell types, such as skin originated dermal fibroblasts, epidermal keratinocytes and immature dendritic cells [30], different human endothelial cells (ECs) including human ECs derived from aortic, coronary artery and saphenous vein [31]. Here, the cell type obtained from tree shrew skin was determined as fibroblasts cells by fluorescence microscopic observation through staining with vimentin antibody (Supplementary Figure S1). Furthermore, the TSVE was determined as endothelial cells, its purity was validated by the presence of CD31/VE-cadherin on cell surface (Supplementary Figure S2). Through the detection for ZIKV RNA and specific protein, as well as the evaluation for the infectiousness of viral progeny in *vivo* and *vitro* (Figure 5), the fibroblasts of tree shrew skin and aorta endothelial cells were proved to be a permissive host cell for ZIKV infection and replication.

In addition, ZIKV infection has resulted in appearance of prominent cytoplasmic effects in TSVE and TSDF (Figure 4). This phenomenon also appeared in human skin fibroblasts with ZIKV infection [30] and other flaviviruses like DENV [32]. This suggested that the skin and vessel of tree shrew might be important sites for ZIKV replication and dissemination, and also seriously damaged by virus [33–35]. Moreover, higher ZIKV-NS1 protein expression was detected in TSVE than that of TSDF cells (Figure 2(C)). Regarding that NS1 is essential for RNA replication of flaviviruses [36], it was confused that two primary cells produced about equivalent viral RNA copies and infectious virus. Taken considered that DENV NS1 may mediated endothelial dysfunction and hyperpermesbility [37], we reason the higher level expression of ZIKV NS1 in TSVE may affect the endothelial integrity.

Previously, we have found that ZIKV-infected tree shrew was induced a strong innate immune response to its infection and characterized by mass hemorrhage with abundant infiltration of inflammatory cells in hypodermis. To understand the specific immune response induced in ZIKV-infected tree shrew *in vitro*, we further assayed the genes expression of some key inflammatory cytokines in the ZIKV infected cells. Our data revealed a strong antiviral immune defence to ZIKV infection in TSVE and TSDF cells. In details, mRNA expression of IL-6, IL-8, CXCL9 and MX1 in infected TSDF was significantly increased. The high levels of these pro-inflammatory chemokine may help to active and recruit immune cells to the sites of infection that contribute to skin toxicity [38–40]. Meanwhile, TNF-α′which induce endothelial barrier dysfunction [41], also has higher levels in TSVE than that of TSDF. Finally, the IFN-β which can trigger warning signals to adjacent cells and induce of cellular antiviral response also had a moderate increases. Collectively, these results showed ZIKV infection have induced a strong immune response *in vitro*.

Taken together, the results presented in this study have demonstrated the skin and the artery can be served as the tropism tissues and support the replication of ZIKV in tree shrew body, and further induce the antiviral immune response to viral infection, which permits us to gain better insight into the new ZIKV infection animal model of tree shrew. Also, the results revealed that primary TSVE and TSDF may serve as a cell model to elucidate the pathogenesis of rash in adult patients.

## Materials and methods

### Experimental animals and ethics statement

Chinese tree shrews (F1 generation) were obtained from the experimental animal core facility of the Kunming Institute of Zoology, Chinese Academy of Sciences. Sprague–Dawley rats and the 1-day-old suckling BALB/C mice were obtained from the Kunming Medical University. To confirm the infectivity of progeny virus, group of 10 1-old-day mice were inoculated by the intracerebral route with 10^3^ PFU of the supernatants or PBS, and monitored daily for morbidity and mortality for 20 days. Two mice in each group were sacrificed on days 5 and 10 to quantify the ZIKV RNAs by using real-time RT-PCR. All experimental procedures and animal care were performed according to the protocols approved by the Institutional Animal Care and Use Committee of the Kunming Institute of Zoology, Chinese Academy of Sciences. The study protocol was reviewed and approved by the Institutional Animal Care and Use Committee of Kunming Institute of Zoology, Chinese Academy of Sciences.

### Virus and cells

ZIKV strain GZ01 (GenBank number KU820898) was isolated from a Chinese male patient returned from Venezuela [42]. Viral culture was performed in Aedes albopictus C6/36 cells and titrated by plaque forming assay on BHK-21 cells [8,43]. HEK293, Huh7.5.1, HEL and HFF-1 cells were cultured in DMEM with L-Glutamine and 10% FBS. HUVEC cells were grown in RPIM-1640 medium (Gibco, USA). The primary cells were isolated from the Chinese Tree Shrews aged from 3 to 4 months which were euthanized by infusion of pentobarbital. The primary hepatocytes were prepared by two-step collagenase perfusion [44] to get the primary hepatocytes cells (TSHC). The different tissues including kidney, lung, skin and the thoracic aorta, were collected to isolate the primary cells as described previously [45–47]. Primary rat skin and aorta cells were isolated and cultured in similar protocol to the Tree Shrews. Contaminating fibroblasts were separated from epithelial cells by negative panning and by several rounds of differential trypsinization to enrich cultures for more adherent epithelial cells.

The different cells were inoculated with one multiplicity of infection of the ZIKV for 1 h and then washed by phosphate-buffered saline (Gibco, USA) to remove the non-attached virus. The infected cells were cultured in minimum essential medium with 2% FBS. All cells were maintained at 37°C with 5% CO2. Experiments involving infectious virus were conducted in a Biosafety level 2 laboratory.

### Detection of ZIKV RNA in supernatants and intracellular of the infected cells

The viral RNA was extracted from cell-culture supernatants using the the PureLink® RNA Mini Kit (Life technologies, USA) and the total RNA of intracellular was using Trizol reagent (Invitrogen) according to the manufacturer’s instructions. Using virus-specific primers and probe were described previously [8], RT-qPCR was carried out with the One-Step PrimeScriptTM RT-PCR Kit (Takara, Dalian, China). For quantification of ZIKV genomic RNA of both positive- and negative-strands in different cells, the intracellular total RNA by strand-specific RT-PCR using the 5’-tagged forward (ZIKV-ASF-Tag) and reverse (ZIKV-ASR-Tag) primers as described previously [8]. Quantitative PCR (qPCR) was then performed with the specific primers and probe for strand specific RNA detection. All the experiments were performed with the CFX Connect™ Real-Time PCR Detection System (Bio-Rad)

### Cell viability assays and Western blotting

The cell viability of infected cells was determined by using Cell Counting Kit-8 (CCK-8) (Sigma-Aldrich, St. Louis, MO) according to the manufacturer’s recommendation. Cell lysates were prepared using a protein lysis buffer (Beyotime Biotech Co.). The protein concentration was determined using a protein assay reagent (Bio-Rad). The obtained protein was separated by sodium dodecyl sulphate polyacrylamide gel electrophoresis and transferred to a polyvinylidene difluoride membrane (Roche Diagnostics). The membrane was blocked with skimmed milk and then incubated with primary antibodies against NS1 (In-house antibody) and GAPDH (Abcam, California, USA), followed by incubation with peroxidase-conjugated anti-mouse IgG (KPL, Inc.). The immunoreactive epitopes were visualized using an enhanced chemiluminescence Western blot detection kit (Millipore).

### Immunofluorescence assay

At different time of ZIKV post-infection, cells were fixed with cold acetone–methanol and saturated with bovine serum albumin. Samples were stained with ZIKV human convalescence serum in blocking solution overnight. Cells were then washed with PBS and incubated with anti-human FITC secondary Abs (Abcam, California, USA) and then incubated with 4, 6-diamidino-2-phenylindole (DAPI) (Thermo) for cell nucleus visualization to allow direct quantification of infection levels. Fluorescence images were acquired on a fluorescence microscope. The number of infected host cells was counted in at least 100 host cells per duplicate. The percentage of infection was estimated by counting the number of infected host cells/number of total host cells. The results were expressed as the mean ± standard deviation of the infection percentage (%).

### Expression analysis of cytokines

The expression of cytokines in the cell lysates were measured at different time of post infection by qRT-PCR, with GAPDH as a housekeeping control gene. Total RNA of cells were extracted using TRIZOL reagent (Life Technologies). The relative RNA levels were quantified using a NanoDrop 2000 spectrophotometer (NanoDrop; Thermo Fisher Scientific, Wilmington, DE, USA). Subsequently, 1 μg RNA was used as a template for the generation of cDNA High-Capacity cDNA Archive Kit (Applied Biosystems), according to the manufacturer’s instructions. Expression of different cytokines mRNAs were measured using SYBR Green (Applied Biosystems). RT-qPCR primer sequences are available on request (see Supplementary Table S4). All reactions were performed using the CFX Connect™ Real-Time PCR Detection System (Bio-Rad). For fold-change calculation of gene copy numbers, we calculated the normalized ratio in uninfected (control) and infected groups by dividing the target gene copy number by the reference gene copy number. Relative gene expression levels were normalized to GAPDH according to the comparative Ct (ΔΔCt) method. Results are expressed as Mean ± SD. Data were analysed using the GraphPad Prism software (GraphPad Software, San Diego, CA, USA).
